# Hypoxia suppresses glucose-induced increases in collective cell migration in vascular endothelial cell monolayers

**DOI:** 10.1038/s41598-024-55706-1

**Published:** 2024-03-02

**Authors:** Kazuki Sone, Yuka Sakamaki, Satomi Hirose, Mai Inagaki, Masanori Tachikawa, Daisuke Yoshino, Kenichi Funamoto

**Affiliations:** 1 Graduate School of Biomedical Engineering, Tohoku University, 6-6-12 Aramaki-aza Aoba, Aoba-ku, Sendai, Miyagi 980-8579 Japan; 2 Institute of Fluid Science, Tohoku University, 2-1-1 Katahira, Aoba-ku, Sendai, Miyagi 980-8577 Japan; 3 Graduate School of Pharmaceutical Sciences, Tokushima University, 1-78-1 Sho-machi, Tokushima, Tokushima 770-8505 Japan; 4 Graduate School of Biomedical Sciences, Tokushima University, 1-78-1 Sho-machi, Tokushima, Tokushima 770-8505 Japan; 5 Institute of Engineering, Tokyo University of Agriculture and Technology, 2-24-16 Naka-cho, Koganei, Tokyo 184-8588 Japan; 6 Graduate School of Engineering, Tohoku University, 6-6-1 Aramaki-aza Aoba, Aoba-ku, Sendai, Miyagi 980-8597 Japan

**Keywords:** Vascular endothelial cell, Cell migration, Glucose, Hypoxia, Microfluidic device, Biomedical engineering, Cell migration, Mechanisms of disease, Diabetes

## Abstract

Blood glucose levels fluctuate during daily life, and the oxygen concentration is low compared to the atmosphere. Vascular endothelial cells (ECs) maintain vascular homeostasis by sensing changes in glucose and oxygen concentrations, resulting in collective migration. However, the behaviors of ECs in response to high-glucose and hypoxic environments and the underlying mechanisms remain unclear. In this study, we investigated the collective migration of ECs simultaneously stimulated by changes in glucose and oxygen concentrations. Cell migration in EC monolayer formed inside the media channels of microfluidic devices was observed while varying the glucose and oxygen concentrations. The cell migration increased with increasing glucose concentration under normoxic condition but decreased under hypoxic condition, even in the presence of high glucose levels. In addition, inhibition of mitochondrial function reduced the cell migration regardless of glucose and oxygen concentrations. Thus, oxygen had a greater impact on cell migration than glucose, and aerobic energy production in mitochondria plays an important mechanistic role. These results provide new insights regarding vascular homeostasis relative to glucose and oxygen concentration changes.

## Introduction

Glucose is the primary source of energy for living organisms. Once incorporated into cells, glucose is converted into pyruvate and lactate acid by cytosolic glycolysis^1^. These intermediates are then used to generate ATP in the mitochondria by aerobic metabolism using oxygen^2,3^. The glucose concentration in blood (blood glucose level) fluctuates daily, changing as much as twofold in a day even in healthy people^4^. A state of high blood glucose level is defined as hyperglycemia, which can lead to diabetes and, in turn, vascular disease. In addition, as cells consume oxygen, the in vivo environment is hypoxic compared to the atmosphere, which is 21% O_2_. For example, the oxygen concentration is only 13.2% even in oxygen-rich arterial blood, and it drops to 5.3% in venous blood^5^. In the cellular response to such hypoxic conditions, hypoxia-inducible factors (HIFs) are expressed and function as intracellular transcription factors^6,7^. Under hypoxic environments, HIF-1α accumulates in cells and translocates to the nucleus, where it induces the expression and secretion of vascular endothelial growth factor (VEGF) and other substances that promote angiogenesis^8^. It has been pointed out that not only the hyperglycemic environment but also the hypoxic environment in vivo plays important roles in the progression of diabetes and its complications^9,10^.

Nutrients such as glucose and oxygen are transported to tissues and cells throughout the body via the blood circulation and vasculature. Vascular endothelial cells (ECs), which cover the lumen of blood vessels in a monolayer, are continuously exposed to mechanical stimulation due to exposure to blood flow, as well as exposed to chemical stimulation from factors in the blood (e.g., glucose, VEGF). Moreover, ECs are affected by temporal changes in the oxygen concentration in the blood, which generates reactive oxygen species (ROS)^11,12^. ROS can be treated by the cells to maintain vascular functions, but excessive ROS could damage blood vessels and cause various vascular diseases^11,13^. ECs contribute to homeostasis by sensing and responding to these environmental factors. ECs in a monolayer migrate by forming small cell groups^14,15^. ECs maintain tissue integrity during remodeling by collectively migrating as a sheet while maintaining appropriate cell distribution^16^. Such collective migration of ECs plays important roles in vascular homeostasis, angiogenesis, and vasculogenesis, as well as tissue regeneration^17–19^ and tumor progression^20,21^. The collective migration of cells is thought to depend on cell–cell and cell–extracellular matrix (ECM) integrity, especially with regard to VE-cadherin^22,23^ for cell–cell adhesion and paxillin^24^ for adhesion between cells and the ECM. With regard to the responses to changes in glucose and oxygen concentrations, wound-healing assays revealed that EC migration decreases under hyperglycemic conditions^25^ and increases under hypoxic conditions^26^. Those results were derived from experiments focusing on the regeneration of a scraped portion of an EC layer rather than an intact monolayer. In addition, exposure of cells to high-glucose (HG) conditions reportedly produce ROS^27,28^ and promote autophagy in ECs^27^. However, most studies related to exposure of ECs to high glucose levels have not considered the in vivo microenvironment oxygen concentration for simplicity. Therefore, the combined effects of local glucose and oxygen concentrations on cellular dynamics, such as changes in migration of ECs in a monolayer, and the underlying mechanisms remain unknown.

A number of recent studies have used microfluidic devices for in vitro cellular research. By culturing cells in micro-size channels fabricated from transparent and highly gas-permeable polydimethylsiloxane (PDMS), it is possible to observe cellular dynamics in real-time while precisely controlling environmental factors around the cells^29–32^. Microfluidic devices that enable oxygen concentration control have also been developed to reproduce in vivo hypoxic conditions^29,30^. We have developed original devices for oxygen concentration control through gas exchange by supplying gas mixtures with a pre-adjusted oxygen concentration^33–36^. Compared to conventional cell experimental methods using cell culture dishes and well plates, microfluidic devices yield precise and fast control of oxygen concentration around cells. By applying these microfluidic devices to study the hypoxic response of ECs, we found that collective cell migration was dependent on oxygen concentration, with the migration speed having a local maximum at approximately 1.7% O_2_, but the speed decreased at lower oxygen concentrations^37–39^. The increase of migration speed was caused by an internalization of VE-cadherin, but the mechanism for the decrease of migration at extremely low oxygen concentrations below 1% O_2_ has not been clarified yet. Additionally, the glucose concentration was that of normally used for cell culture (5.5 mM). Thus, the changes in cell migration caused by the combined effects of two environmental factors, glucose and oxygen concentrations, remain unclear.

**Figure 1:**
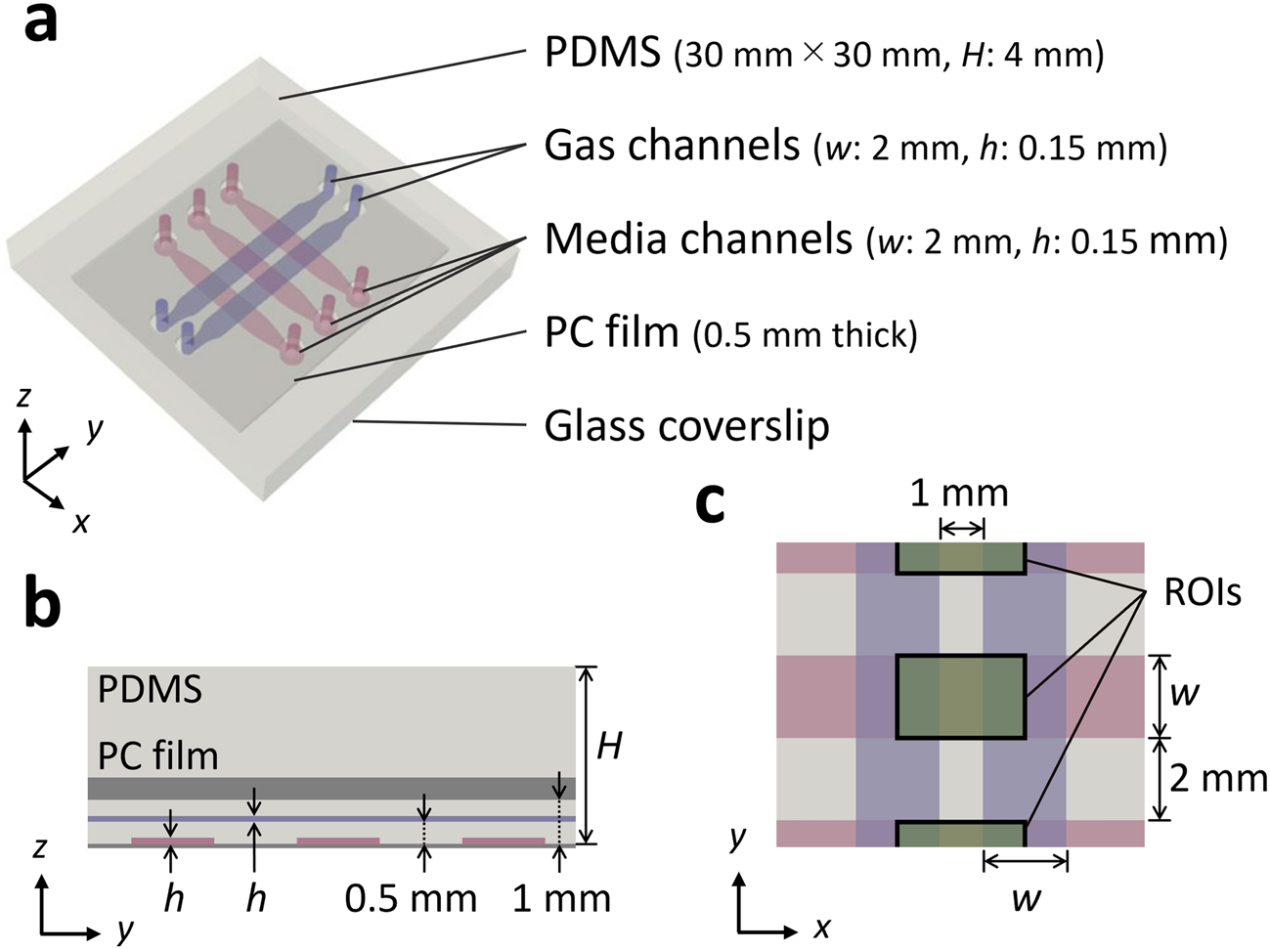
Microfluidic device used for cellular experiments. (**a**) Perspective view, (**b**) enlarged cross-sectional view in the *yz*-plane, and (**c**) enlargement of the channels in the *xy*-plane. ROIs were set in the media channels between the gas channels.

In this study, we evaluated the early-stage changes in cell migration resulting from variations in pericellular glucose and oxygen concentrations and discussed the underlying mechanism. An EC monolayer was formed in the media channels of a microfluidic device in which the oxygen concentration could be controlled, and cell culture medium at a controlled glucose concentration was supplied to the media channels (Fig. 1). Simultaneously, the oxygen concentration in the cell culture medium was controlled by supplying the gas channels with gas mixture containing a preadjusted oxygen concentration. Time-lapse phase-contrast images of the EC monolayer were then obtained and the cell migration velocity was measured by particle image velocimetry (PIV), in which the images were subdivided and the displacement of each subdomain was calculated by cross-correlation function between the sequential images. Expression of the factors VE-cadherin and HIF-1α, which can affect the cell migration, were also evaluated by immunofluorescence staining to assess their expression levels and localization. Furthermore, focusing on aerobic metabolism as a possible mechanism that affects cell migration, the effect of mitochondrial energy production on cell migration was examined in experiments using mitochondrial electron transport inhibitors as well as by quantifying cellular ATP production.

## Results

### Combined effects of glucose and oxygen on cell migration

**Figure 2:**
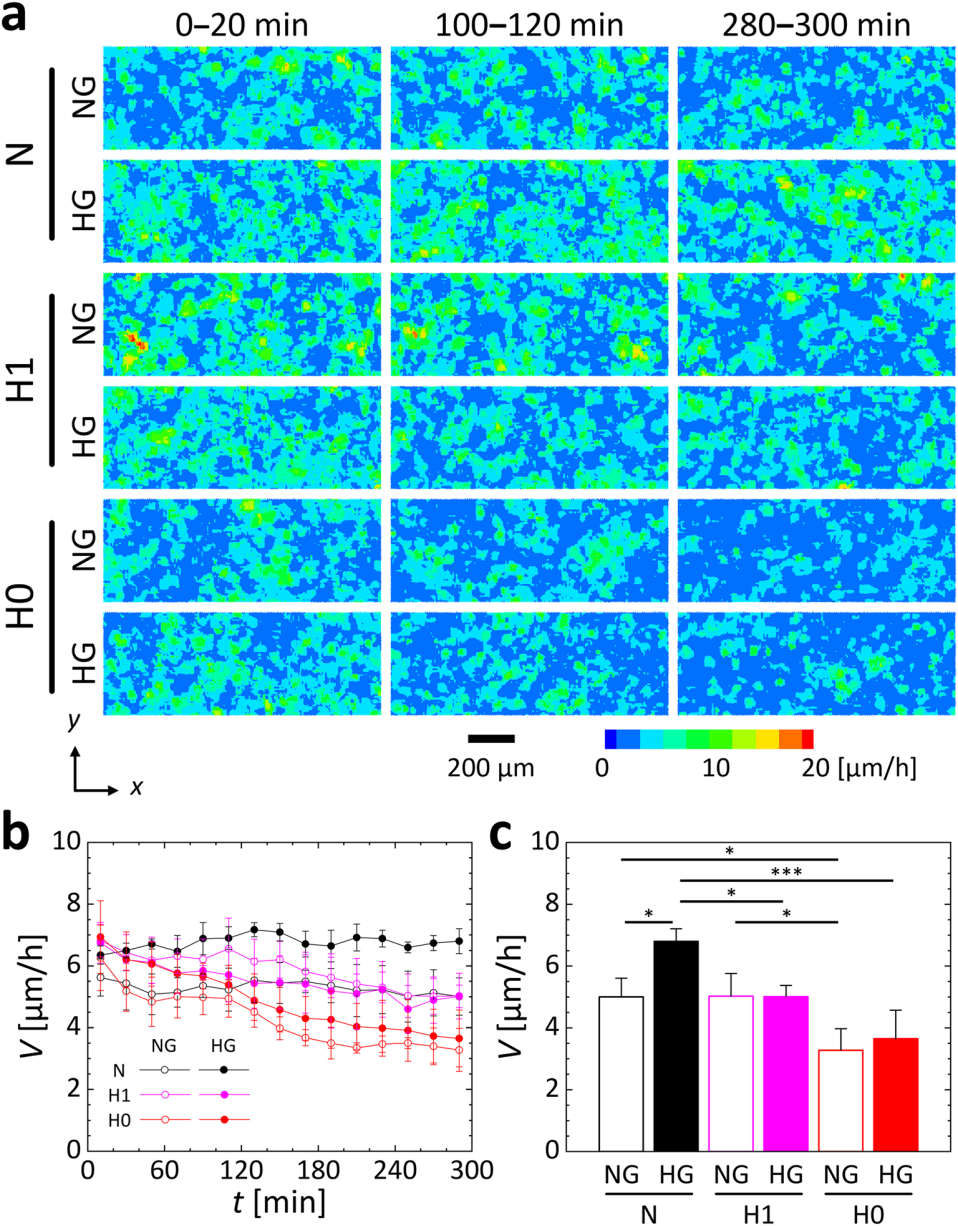
Collective migration of HUVECs under normal or high D-glucose conditions at 5.5 mM (NG) or 30 mM (HG) and three oxygen conditions with supply of gas mixtures at 21%, 1%, or 0% O_2_ (N, H1, or H0, respectively). (**a**) Contour map of migration speed obtained by PIV analysis using phase-contrast microscopy images at 20-min intervals. (**b**) Average migration speed, *V*, over time for 5 h, and (**c**) the average migration speed at 5 h. Error bars show the standard deviation. Significant differences in migration speed between the glucose and oxygen conditions were assessed by two-way ANOVA followed by Tukey’s post-hoc test for multiple comparisons. ^*^P < 0.05; ^***^P < 0.001.

An EC monolayer was formed on the bottom of the media channels of a microfluidic device, and collective migration of the cells was then observed. Migration velocity data obtained using PIV analysis with time-series phase-contrast microscopy imaging at the intervals of 20 min showed that the cells migrated randomly, forming small clusters (Fig. 2a). Regions exhibiting a high migration speed always became wider under normoxic HG condition. In contrast, under hypoxic conditions H1 and H0, the changes in migration speed with changing glucose concentration were small. In particular, under hypoxic condition H0, the region exhibiting a high migration speed diminished over time. The spatial average migration speed was almost constant throughout the experimental period under normoxic condition for each glucose concentration (Fig. 2b). Under hypoxic condition H1, migration speed showed a tendency of increase compared to the normoxic normal glucose (NG) condition during the first 2 h of the experiment. However, it started to decline thereafter. In contrast, migration speed exhibited a continuous decrease from the beginning of the experiment under HG conditions. In the both glucose cases under hypoxic condition H1, the migration speed converged to the same level as that under normoxic NG condition after 5 h. Under hypoxic H0 condition, the migration speed decreased immediately after the start of the experiment under the NG condition, showing an almost constant value until 2 h, decreasing thereafter. Basically, the migration speed decreased continuously at both glucose concentrations, but it tended to be always greater under HG conditions. Comparison of the migration speed at 5 h indicated that, for normoxic condition N, the migration speed was greater under the HG condition than under the NG condition (Fig. 2c). In contrast, under hypoxic conditions H1 and H0, the variations in migration speed with changing glucose concentration were small. Regardless of glucose conditions, the migration speed decreased under hypoxic condition H0. As described above, hypoxia suppressed the enhancement of migration caused by high glucose, and decreased the migration speed. Here, regardless of glucose or oxygen conditions, the increase rate in cell numbers, *α*_I_, hardly changed after each experiment, showing a value slightly larger than 1 (Fig. S1). This indicates that the EC monolayer was confluent and maintained an almost constant density throughout the experiment.

The effect of the change in osmotic pressure resulting from the increase in glucose concentration was investigated by measuring the migration speed following the addition of L-glucose (L-glu) to the cell culture medium instead of D-glucose (D-glu). The addition of L-glu at the beginning caused a similar increase in the migration speed as the addition of D-glu (Fig. S2a). However, after 2 h, the migration speed declined and approached that observed under the NG condition. Although the spatial average migration speed at 5 h under the HG condition with L-glu supplementation tended to increase compared with the NG condition (no significant difference), the increase was smaller than that observed under the HG condition with D-glu supplementation (Fig. S2b). Thus, changes in osmotic pressure affect the cell migration speed just after glucose supplementation, but this effect diminishes after several hours. We also investigated changes in migration speed with changes in the D-glu concentration (Fig. S3). The migration speed increased with increasing D-glu concentration, but it reached a plateau at concentrations > 15 mM, showing no difference at 5 h at a D-glu concentration ≥ 22.5 mM (Fig. S3b).

Moreover, the size of clusters in collective migration of ECs was evaluated by calculating the autocorrelation function *C*_*vv*_ of the velocity fluctuation vectors δ**v** obtained by subtracting the spatial average velocity $\overline{\mathbf{v} }$ from the velocity vector **v** as the following formula:

$$C_{vv} = \left\langle {\frac{{\mathop \sum \nolimits_{i} {\updelta }{\mathbf{v}}\left( {{\mathbf{r}}_{i} } \right) \cdot {\updelta }{\mathbf{v}}\left( {{\mathbf{r}}_{i} + {\mathbf{r}}} \right)}}{{\mathop \sum \nolimits_{i} {\updelta }{\mathbf{v}}\left( {{\mathbf{r}}_{i} } \right) \cdot {\updelta }{\mathbf{v}}\left( {{\mathbf{r}}_{i} } \right)}}} \right\rangle ,$$

where **r**_*i*_ is the position where velocity vector of cell migration was measured, and the angle brackets denote an average over all direction and time^38,40, 41^. It was observed that the autocorrelation function decreased exponentially under each condition (Fig. S4). The cluster size tended to increase by HG condition and to decrease under hypoxic condition.

**Figure 3:**
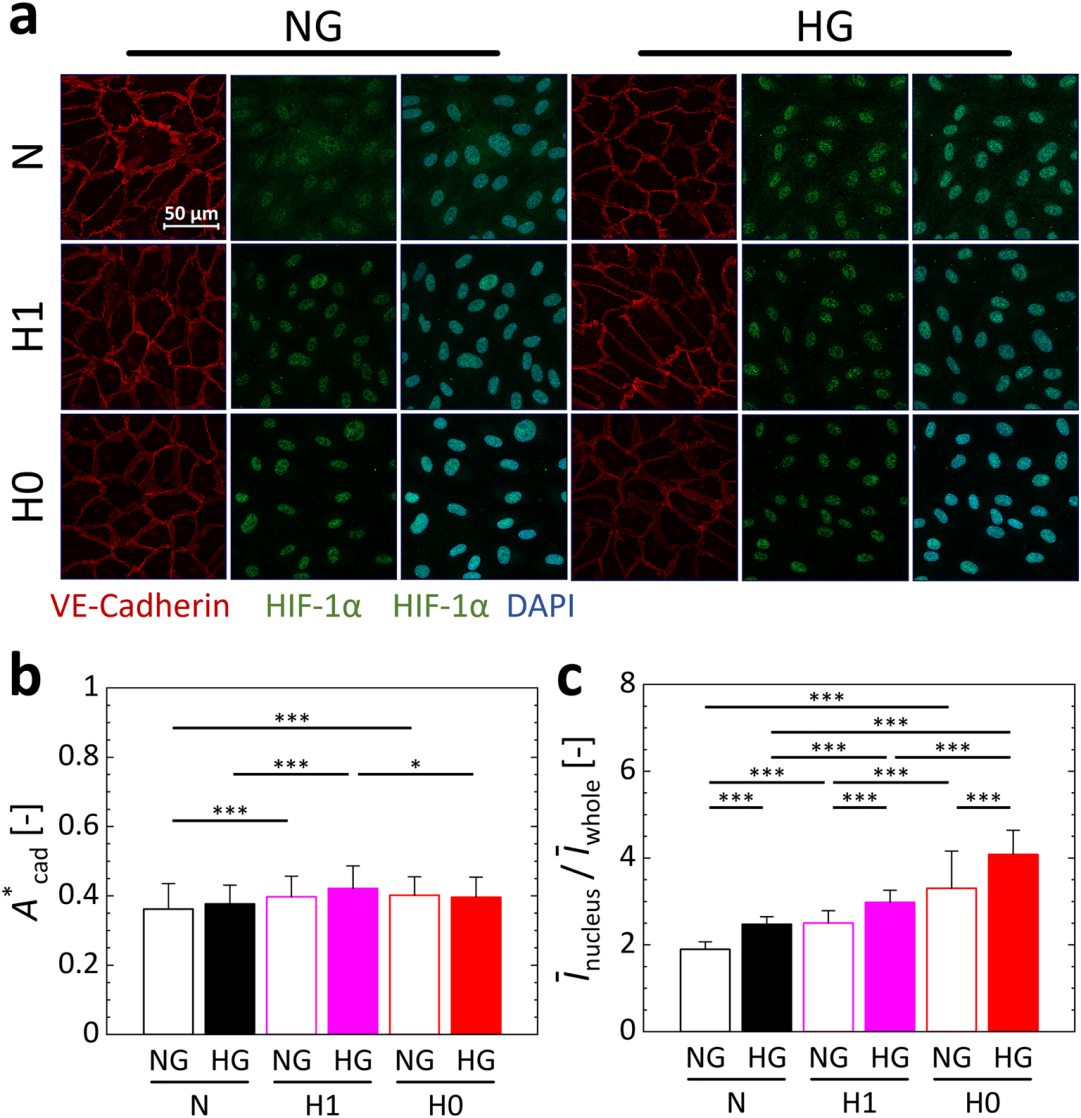
Changes in intracellular protein expression and localization in HUVECs under normal or high D-glucose conditions of 5.5 mM (NG) or 30 mM (HG) and three oxygen conditions with supply of gas mixtures at 21%, 1%, or 0% O_2_ (N, H1, or H0, respectively). (**a**) Representative images of maximum-intensity projections of confocal microscopy images of VE-cadherin (red), HIF-1α (green), and nuclei (DAPI, blue) in HUVECs relative to the *xy*-plane. (**b**) Relative area of VE-cadherin to the total cell area, A^*^_cad_. (**c**) Ratio of the mean fluorescence intensity of HIF-1α in the nucleus to that in the whole cell, ${\overline{I} }_{{\text{nucleus}}}/{\overline{I} }_{{\text{whole}}}$. Error bars show the standard deviation. Significant differences between the glucose and oxygen conditions were assessed by two-way ANOVA followed by Tukey’s post-hoc test for multiple comparisons. ^*^P < 0.05.

Next, the expression and localization of VE-cadherin and HIF-1α were examined using immunofluorescence staining. The maximum intensity projection of *z*-stack confocal microscopy images of cells indicated that VE-cadherin localized along the cellular edge, but no clear differences were observed under the different experimental conditions (Fig. 3a). The relative area of VE-cadherin to the whole cell, A^*^_cad_, showed little difference by high-glucose exposure under each oxygen condition (Fig. 3b). Hypoxic exposure increased A^*^_cad_ under NG condition, and hypoxic condition H1 maximized A^*^_cad_ under HG condition. HIF-1α translocated to the nucleus as the glucose concentration increased or the oxygen concentration decreased (Fig. 3a), increasing the nuclear translocation rate of HIF-1α, ${\overline{I} }_{{\text{nucleus}}}/{\overline{I} }_{{\text{whole}}}$, (Fig. 3c).

### Dependence of migration on mitochondrial function

**Figure 4:**
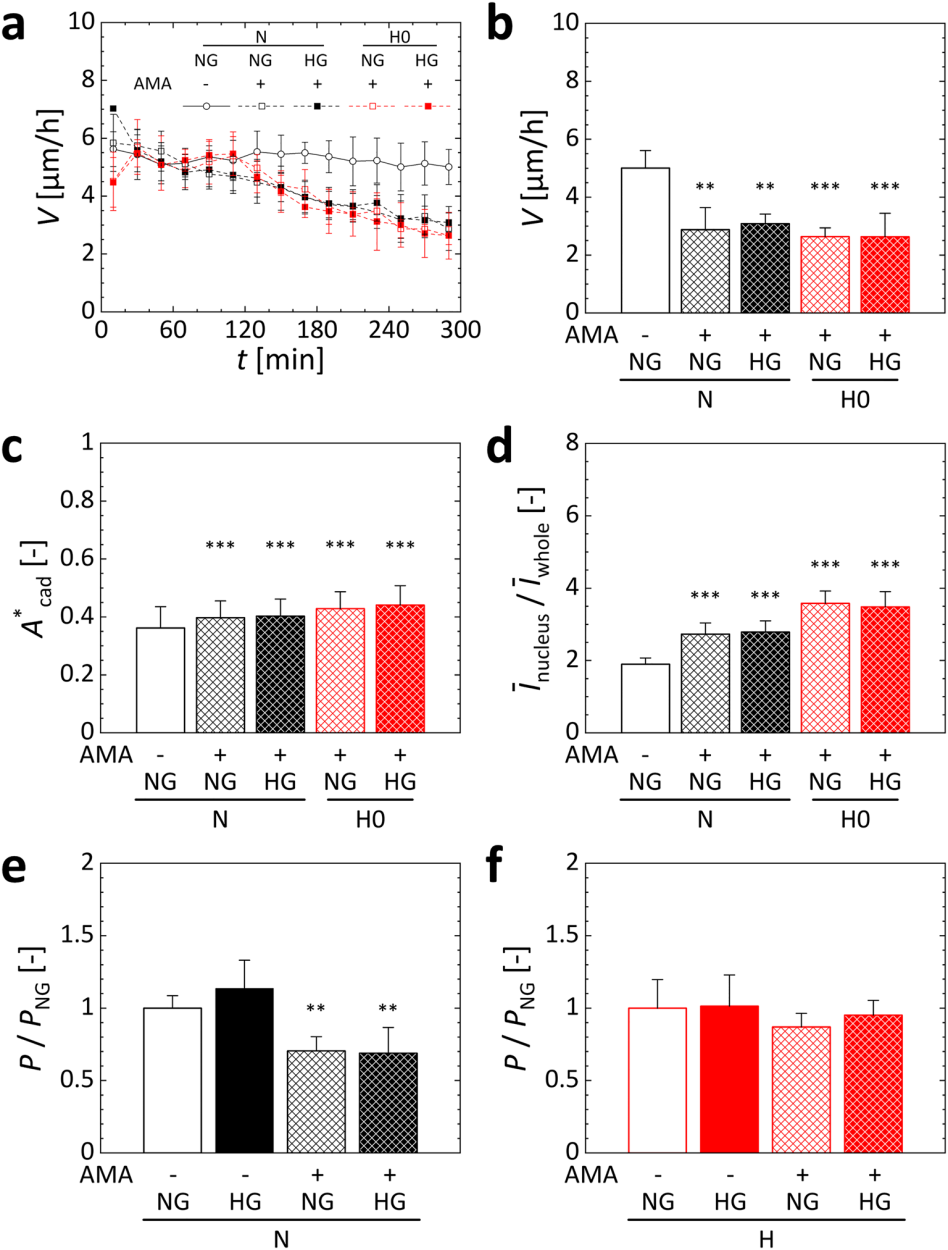
Changes in collective migration of HUVECs, intracellular protein expression and localization, and intracellular ATP production under normal or high D-glucose conditions of 5.5 mM (NG) or 30 mM (HG) and normoxic (21% O_2_, N) or hypoxic (0.3% O_2_, H0; 0.1% O_2_, H) conditions with inhibition of mitochondrial electron transport by AMA. (**a**) Average migration speed, *V*, over 5 h, and (**b**) the average migration speed at 5 h. (**c**) Relative area of VE-cadherin to whole cell area, A^*^_cad_. (**d**) Ratio of the mean fluorescence intensity of HIF-1α in the nucleus to that in the whole cell, ${\overline{I} }_{{\text{nucleus}}}/{\overline{I} }_{{\text{whole}}}$. Normalized values of intracellular ATP under (**e**) normoxic and (**f**) hypoxic conditions. The amount of ATP produced was normalized to that produced under the NG condition without AMA in the same cell culture plate, as *P*/*P*_NG_. Error bars show the standard deviation. Significant differences were assessed by one-way ANOVA followed by Tukey’s post-hoc test for multiple comparisons. ^**^P < 0.01; ^***^P < 0.001 versus normoxic NG condition without AMA.

Focusing on aerobic metabolism as a possible mechanism which leads changes of cell migration by glucose and oxygen conditions, the effect of mitochondrial metabolism on cell migration was further examined. The cell migration was observed under each glucose and oxygen condition by supplementing the cell culture medium with antimycin A (AMA), an inhibitor of mitochondrial electron transport (Fig. S5a). Under either glucose and oxygen concentration condition, collective cell migration gradually decreased just after starting the experiments. The migration speed of the cells continuously decreased following the addition of AMA (Fig. 4a), in contrast to the almost constant speed under normoxic condition N without AMA addition. Under hypoxic condition H0, the migration speed was almost constant for approximately 2 h after AMA was added but then decreased to be the same level as the other conditions supplemented with AMA. The migration speed of cells at 5 h after AMA supplementation was almost the same regardless of glucose conditions (Fig. 4b). The autocorrelation function *C*_*vv*_ of the velocity fluctuation vectors δ**v** of ECs showed that the size of clusters in collective migration decreased by AMA, especially under hypoxic HG condition (Fig. S6).

The expression and localization of VE-cadherin and HIF-1α were also examined by immunofluorescence staining of cells supplemented with AMA (Fig. S5b). Although differences were difficult to detect in images of VE-cadherin staining, the relative area of VE-cadherin to the total cell area, A^*^_cad_, increased by AMA and tended to increase more under hypoxic condition (Fig. 4c). Nuclear translocation of HIF-1α was observed after exposing the cells to high-glucose or hypoxia, even with AMA supplementation (Fig. S5b). The nuclear translocation rate of HIF-1α, ${\overline{I} }_{{\text{nucleus}}}/{\overline{I} }_{{\text{whole}}}$, increased approximately 1.5- and twofold under normoxic condition N and hypoxic condition H0, respectively, following addition of AMA (Fig. 4d). It should be noted that under each oxygen conditions, increasing glucose had no effect on the nuclear translocation rate of HIF-1α, different from the promotion of nuclear translocation by high glucose as described in the previous section.

Intracellular ATP production was evaluated separately by extracting ATP from cells cultured in 96-well cell culture plates and measuring the level using the firefly luciferase luminescence method. Under normoxia, ATP production increased under the HG condition compared to the NG condition but decreased following supplementation with AMA, regardless of glucose concentration (Fig. 4e). On the other hand, under the hypoxia of 0.1% O_2_ generated using a hypoxic box^42^, changing the glucose concentration had little impact on ATP production, as the level was similar under all conditions (Fig. 4f).

## Discussion

The collective migration of ECs changes depending on the glucose and oxygen concentrations around the cells, with the latter having a greater effect. Cell migration is increased by exposure to HG condition but decreased by simultaneous exposure to hypoxia. This variation in cell migration involves aerobic energy metabolism in mitochondria.

The glucose and oxygen conditions had no effect on cell viability (Fig. S1), suggesting that the increase or decrease in migration is not due to changes in viability and cell density. Additionally, the timing of the stimulation had little effect on cell migration^43^. Under oxygen-rich normoxic condition, cell migration increased with increasing glucose concentration in the cell culture medium (Figs. 2 and S3). Measurement of cell migration at different concentrations of D-glu showed that the migration speed increased with increasing D-glu concentration, but the speed were similar at concentrations > 15 mM (Fig. S3). An increase in cell migration speed resulting from high-glucose exposure was observed just after exchanging the cell culture medium with high-glucose medium and lasted for at least 3 days^43^. Therefore, cells were exposed to HG conditions just before time-lapse observation of cell migration by replacing the cell culture medium with medium containing an adjusted D-glu concentration of 30 mM, at which cell migration clearly changed.

The increase in cell migration speed induced by high glucose could be caused by glucose-related changes in the osmotic pressure of the cell culture medium. As such, instead of D-glu, we added L-glu, which is not used by cells for energy metabolism, to generate an alternative HG condition and then examined changes in cell migration. The cell migration speed increased for about 2 h following the addition of L-glu but then decreased and approached the rate observed at a normal glucose concentration (Fig. S2). In other words, an increase in osmotic pressure promotes cell migration, but the effect is temporary, subsiding within a few hours. In the present study, the cell migration speed continued to increase for 5 h after exposure to the HG condition (Fig. S3). In a previous study^43^, a similar increase in cell migration speed was observed after 3 days of exposure to HG conditions. Thus, the change in osmotic pressure due to high D-glu concentrations seems to have little effect. In addition, under hypoxic condition H1, the migration speed after approximately 2 h tended to be lower under the HG condition than the NG condition (Fig. 2b). Consequently, the effect of osmotic pressure is suppressed by exposure to hypoxia, suggesting that oxygen concentration has a greater impact on cell migration than osmotic pressure.

Under hypoxic conditions, the increase in cell migration speed induced by high-glucose exposure was suppressed (Fig. 2). The level of intracellular ATP increased under the normoxic HG condition compared to the NG condition, but there was no difference between the glucose conditions under hypoxia (Fig. 4e,f). This trend was consistent with the changes in migration speed (Fig. 2). Regarding the relationships between ATP and cell dynamics, a decrease in intracellular ATP reportedly induces cellular autophagy^44^ and suppresses cancer cell migration^45^. Moreover, as aerobic metabolism is more energy efficient than anaerobic metabolism, the oxygen concentration in the environment surrounding the cells can have a greater effect on ATP production than the surrounding glucose concentration. These results suggest that ATP production is also involved in changes in cell migration speed and that the effect of the oxygen concentration around the cells is greater than that of the glucose concentration. Previous studies on wound-healing have reported that cell migration decreases with increasing glucose concentration^25^, but this study revealed for the first time that high glucose increases collective cell migration in an intact monolayer, which is suppressed by hypoxia.

By inhibiting mitochondrial electron transport with AMA, the cell migration speed decreased under all glucose and oxygen conditions, resulting in similar values (Fig. 4a,b). In this study, AMA solution containing ethanol as a solvent was added to the cell culture medium. Our previous study revealed that ethanol diluted 1,250-fold with cell culture medium increased migration^38^. The ethanol in the cell culture medium in this study was diluted 4,000-fold, and the addition of AMA effectively reduced cell migration (Fig. 4a,b). Hence, the effect of the solvent ethanol was considered negligible. The amount of intracellular ATP decreased following treatment with AMA under normoxic conditions, but there was no difference between the NG and HG conditions (Fig. 4e,f). However, under hypoxic conditions, the change induced by AMA was small, consistent with the trend in the change in the cell migration speed (Fig. 4b). These data suggest that aerobic energy production in mitochondria governs the cell migration speed.

The relative area of VE-cadherin, which plays a role in intercellular adhesion, tended to increase under hypoxic conditions H1 or H0 and under AMA addition conditions (Figs. 3b and 4c). Additionally, the migration speed shows a declining trend under these conditions (Figs. 2c and 4b). Our previous study also showed that cell migration decreases with increased VE-cadherin expression^38^ and increases following internalization of VE-cadherin induced by hypoxia^37–39^. On the other hand, though there was little difference in the relative area of VE-cadherin by glucose conditions under each oxygen condition, the migration was increased by high-glucose exposure under normoxic condition (Figs. 2c and 3b). This result suggests that internalization of VE-cadherin is not involved in the increase in migration speed induced by high-glucose exposure and that one or more other factors may be involved. In summary, the cell–cell adhesion mediated by VE-cadherin could be responsive to migration speed change by hypoxia, but not by high-glucose exposure.

The previous study found that migration speed increased as the oxygen concentration decreased down to about 1.7% O_2_ due to an internalization of VE-cadherin, but the migration decreased at an extremely low oxygen concentration below 1% O_2_^39^. This study revealed that in an extremely low oxygen concentration environment of 0.3%, migration is reduced by a decrease in aerobic metabolism. These results suggest that the environment with an oxygen concentration of 1.3% created under the present hypoxic condition H1 can be a turning point which changes migration mechanisms between the two phases, resulting in the same level of migration speed as normoxic condition N. Unlike the present result, it has been reported that the expression of VE-cadherin decreases in ECs cultured in high-glucose medium^46^. Previously, we also showed a decrease in the relative area of VE-cadherin after 3 days of high-glucose exposure to ECs, different from the high-glucose exposure for 5 h^43^. These results suggest that expression and localization of VE-cadherin depends on the glucose condition, and changes time-dependently. Here, the cluster size of cell migration increased under HG condition (Fig. S4), which could be caused by the enhancement of cell–cell adhesion forming the monolayer of ECs. Under hypoxic condition H0 or AMA addition condition, an increase in the relative area of VE-cadherin and decreases in the speed and cluster size of collective migration of ECs were simultaneously observed (Figs. 2c, 3b, 4b,c, S4, and S6). Consequently, an enhancement of VE-cadherin might cause the reduction of migration under microenvironment where oxygen is not sufficiently available for metabolism, resulting in small clusters.

The nuclear translocation rate of HIF-1α increased under hypoxic conditions, suggesting that the cells can sense pericellular hypoxia even if the surrounding environment contains a high concentration of glucose (Fig. 3). Under the both oxygen conditions examined, HIF-1α translocated into the nucleus in the presence of high glucose (Fig. 3c). This phenomenon is consistent with the report that HIF-1α is stabilized by hyperglycemia^47^. Incremental increases in glucose concentration increase oxygen consumption by mitochondria, resulting in intracellular hypoxia^48^. Hence, the increased nuclear translocation of HIF-1α under the HG condition examined in this study could have been caused by intracellular hypoxia. Furthermore, inhibition of mitochondrial function with AMA resulted in similar rates of nuclear translocation of HIF-1α under both the NG and HG conditions at each oxygen concentration (Fig. 4d). Inhibition of mitochondrial function is reportedly exacerbated by the generation of ROS^49–51^, and HIF-1α is stabilized by ROS generation^52^. Therefore, the observed increase in nuclear translocation of HIF-1α upon the addition of AMA could have been related to ROS generation within the cells, caused by inhibition of mitochondrial function. In our previous study^39^, we found that the cell migration speed decreased at extremely low oxygen concentrations at which nuclear translocation of HIF-1α markedly increased. Similarly, a simultaneous increase in the nuclear translocation of HIF-1α and decrease in cell migration speed was observed under hypoxic condition H0 or AMA addition condition (Figs. 2c, 3c, 4b,d), indicating a close relationship between those two factors. In addition, under hypoxic conditions regardless of AMA, the migration speed was increased for about 2 h, and then decreased (Figs. 2b and 4a). In our other previous study^38^, the nuclear translocation of HIF-1α in response to exposure to hypoxia showed a temporal variation, reaching the maximum after 2 h. These results suggested that the cellular hypoxic response resulting in high migration speed is related to HIF-1α stabilization. And, after about 2 h, migration could be decreased because of ATP deficiency.

The microfluidic device used in this study has the advantage of minimizing the amount of reagents and cells needed for analyses. However, because of the low sample volume, it was difficult to quantitatively evaluate intracellular proteins. In order to evaluate ATP production by the cells, it was necessary to perform experiments using cells cultured in cell culture plates separately from the experiments using the microfluidic device. The ATP amount obtained in the experiments still varied between the well plates even under the same condition (Fig. S7), and a more accurate method to quantify ATP amount is required. Many details regarding the relationship between changes in migration, intercellular adhesion, and HIF-1α expression also remain unclear, and thus, further investigation of intracellular signal transduction is required. Additionally, mitochondrial function was inhibited by disruption of electron transport. More detailed investigations will be required to elucidate the relationship between cell migration and metabolism using approaches such as inhibition of the citric acid cycle, which is located upstream of the metabolic pathway. Examining the effects of glucose and oxygen on mitochondria function will be also a subject for future research. Although this study conducted experiments with two-dimensional EC monolayers, the physiologically morphological and functional changes, such as angiogenetic events and vascular permeability, will be further investigated by experiments with three-dimensional vascular structure or microvascular network formed in microfluidic devices.

## Conclusion

Changes in the collective migration of ECs by simultaneous exposure to high-glucose concentrations and hypoxia were investigated by cellular experiments using a microfluidic device. Cell migration increased with increasing glucose concentration in a normoxic environment. On the other hand, the migration speed increase was suppressed by exposure to hypoxia, suggesting that the oxygen concentration has a greater effect on cell migration than the glucose concentration. Furthermore, inhibition of mitochondrial electron transport decreased the cell migration speed to a value similar to that observed under severe hypoxic condition. Thus, aerobic energy production by mitochondria has a significant effect on cell migration. These results enhance our understanding of the mechanism by which vascular homeostasis is maintained in response to changes in glucose and oxygen concentrations.

## Methods

### Microfluidic device

The microfluidic device used for oxygen concentration control was fabricated with PDMS, a polycarbonate (PC) film, and a glass coverslip (Fig. 1)^36^. The device was square, with sides of 30 mm, and two gas channels (width 2 mmμ, height 150 μm) were placed at a height of 500 μm, perpendicular to three parallel media channels (width 2 mm, height 150 μm) on the bottom. In order to prevent infusion of oxygen from the atmosphere around the device, a 0.5-mm-thick PC film was embedded at a height of 1 mm. To fabricate the device, PDMS (Sylgard 184 Silicone Elastomer Kit, Dow Corning, USA) was poured to a thickness of 0.5 mm onto each silicon wafer on which the media or gas channel patterns were layered by photolithography, to be 0.5 mm thickness, followed by curing in an oven (60 °C) for more than 4 h. Additional PDMS was then poured onto the cured PDMS layer of the gas channels to a thickness of 2.5 mm, and the above-mentioned PC film with holes opened at the ports of the media and gas channels were submerged and placed. After curing the PDMS in the oven overnight, the PDMS layers of the media channels and gas channels were respectively peeled off from the silicon wafers, cut into squares with sides of 30 mm, and bonded together by plasma treatment. The PDMS mold and glass coverslip were then sterilized by autoclaving and bonded together by plasma treatment. Immediately afterward, 1 mg/ml poly-D-lysine (P7886, Sigma-Aldrich, USA) solution was injected into the media channels, and the device was placed in an incubator (37 °C) overnight. The media channels were then washed twice with sterile water. Before cell seeding, the surface of the media channels was coated with 50 μg/ml fibronectin (FC010, Millipore, USA) by injection to further enhance cell adhesion.

### Cell migration analysis

Human umbilical vein endothelial cells (HUVECs) (C2519A, Lonza, Switzerland) between the 5th and 7th passages were used in the experiments. The cells were cultured on dishes with cell culture medium (EGM-2 BulletKit, CC-3162, Lonza) in an incubator (5% CO_2_, 37 °C) and harvested before reaching confluence. A suspension of 2.5 × 10^6^ cells/ml was then prepared and 25 μl was injected into each media channel of the device. After allowing HUVECs to adhere to the media channels in the incubator for 30 min, the cell culture medium was changed. By culturing the cells for 3 days, changing the cell culture medium every day, a confluent EC monolayer was formed covering the surface of the media channels.

To elucidate the effect of changes in glucose concentration around ECs on migration, the cells were exposed to a HG condition using EGM-2 additionally supplemented with glucose. High-glucose cell culture medium (HG condition) was prepared by adding D-glu (G0048, Tokyo Chemical Industry, Japan) to EGM-2 at a concentration of 15, 22.5, or 30 mM, whereas the original EGM-2 (NG condition) contained 5.5 mM D-glu. The effect of osmotic pressure on cell migration was examined using another high-glucose cell culture medium prepared by adding 24.5 mM L-glu (G0226, Tokyo Chemical Industry), an enantiomer of D-glu, to EGM-2. The cell culture medium in the media channels was replaced with control or high-glucose medium just before observing cell migration^43^.

The contribution of mitochondrial activity on cell migration was investigated using AMA (A8674, Sigma-Aldrich)^53^, an antibiotic produced by the soil microorganism *Streptomyces*^54^. AMA inhibits mitochondrial ATP synthesis by inhibiting electron transfer to ubiquinone in the mitochondrial cytochrome bc1 complex^55^. AMA was dissolved in absolute ethanol (09–0770-4, Sigma-Aldrich) as a solvent to prepare a 100 mM solution, which was further diluted with control or high-glucose cell culture medium to a concentration of 25 µM. The cell culture medium in the media channels was replaced with medium containing AMA just before the experiment.

The device with a confluent EC monolayer was placed in a stage incubator (INUBSF-ZILCS, Tokai Hit, Japan) (5% CO_2_, 37 °C) mounted on an inverted microscope (EVOS FL, Cell Imaging System, Life Technologies, USA). The oxygen concentration in the three media channels was controlled to the same condition by supplying gas mixtures with 21%, 1%, or 0% O_2_, maintaining 5% CO_2_ balanced with nitrogen, to both gas channels using a gas blender (3MFC GAS MIXER, KOFLOC, Japan). Using these gas mixtures, an actual oxygen concentration of 21%, 1.3%, or 0.3% O_2_ was generated in the media channels, respectively (normoxic condition N, hypoxic conditions H1, or H0)^36^, focusing on cell migration under very low oxygen concentrations. By supplying a gas mixture containing 21% O_2_ and 5% CO_2_ to the gas channels for 1 h, a steady normoxic state was first generated, and the temperature and pH in the device were stabilized. Manipulation of the oxygen concentration was initiated by supplying the gas mixture to the gas channels, and time-series phase-contrast microscopy images were obtained every 10 min for 5 h. Microscopy images collected at intervals of 20 min were analyzed using PIV software (JPIV)^56^ to quantify cell migration. A region of interest (ROI) of 1,280 × 512 pixels (1,100 × 440 μm) was set covering the center of the media channels, away from the side walls of the media channels. The cell migration velocity was measured based on the displacement of the subdivided region (8 × 8 pixel or 6.875 × 6.875 µm), comparable to cell size, in the ROI. Another ROI of 128 × 128 pixels (110 × 110 μm) was set outside of the media channels, and the displacement by the deviation of the device itself was measured. Cell migration velocity was then corrected by subtracting the device displacement. The increase rate in cell number or cell density was evaluated using the phase-contrast microscopy images taken before and after the experiment. The cell migration experiments were conducted using four devices for each experimental condition. Significant differences in migration speed were assessed by one-way or two-way analysis of variance (ANOVA) followed by Tukey’s post hoc test, and statistical significance was inferred at* P* < 0.05.

### Immunofluorescence analysis

VE-cadherin and HIF-1α expression in HUVECs was observed by immunofluorescence staining. After time-lapse observation of cell migration, the cells in the media channels were fixed with 4% paraformaldehyde phosphate buffer (163–20,145, Wako Pure Chemical Industries, Japan) for 10 min. Next, the cell membrane was permeabilized with 0.1% Triton X-100 (Pharmacia Biotech, Sweden) for 10 min. The cells were subsequently blocked with 1% Block Ace (DS Pharma Biomedical, Japan) solution diluted with phosphate-buffered saline (PBS) (P5119, Sigma-Aldrich) for 30 min to suppress nonspecific adsorption of antibodies. VE-cadherin and HIF-1α were labeled for 1 h with primary antibodies (sc-9989, Santa Cruz Biotechnology, USA; ab51608, Abcam, USA) diluted 100-fold with PBS, followed by staining with secondary antibodies for 1 h, Alexa Fluor 594 goat anti-mouse antibody (A11032, Invitrogen, USA) and Alexa Fluor 488 goat anti-rabbit antibody (A11008, Invitrogen), each diluted 100-fold with PBS. Nuclei were stained with 5 μg/ml DAPI (D21490, Thermo Fisher Scientific) for 10 min. Immunofluorescence staining was performed at room temperature, and between each step, the cells in the media channels were washed twice with PBS. After immunofluorescence staining, the cells were observed using a confocal laser scanning microscope (LSM800, Carl Zeiss Microscopy, Germany). Twenty cross-sectional images of the cells on the bottom of the device were taken at 0.60-µm intervals in the vertical direction, and an image was created by projecting the maximum fluorescence intensity onto a horizontal plane.

Protein localization and expression were evaluated using the projected images with the image processing software ImageJ (NIH, USA). Cell–cell integrity was quantified based on the relative area of VE-cadherin staining. The relative area of VE-cadherin *A*_cad_ in each cell relative to the total cell area *A*_out_ was calculated as A^*^_cad_ (= *A*_cad_/*A*_out_)^38,39, 43^. Cellular hypoxic responses were assessed by quantifying the nuclear translocation rate of HIF-1α as a starting point of the hypoxic response. Images of HIF-1α nuclear translocation were generated by taking the product of the binarized nucleus images and HIF-1α images. The average fluorescence intensity ${\overline{I} }_{{\text{nucleus}}}$ and ${\overline{I} }_{{\text{whole}}}$ within the nucleus and the entire image was measured, and then the ${\overline{I} }_{{\text{nucleus}}}/{\overline{I} }_{{\text{whole}}}$ ratio was calculated as the nuclear translocation rate of HIF-1α^38,39^.

All microscopic evaluations were performed using four devices for each condition, and nine locations were analyzed in each device (36 locations in total). For the evaluation of VE-cadherin, three cells were randomly chosen in each microscopy image, and 108 cells in total were employed. Significant differences in metrics were assessed by one-way or two-way ANOVA followed by post hoc Tukey’s test, with statistical significance inferred at *P* < 0.05.

### ATP measurement

ATP production by HUVECs under each experimental condition was analyzed using 96-well cell culture plates. The surface of the plate wells was first coated with fibronectin in the same way as the media channels of the microfluidic device. A suspension containing 1.2 × 10^4^ HUVECs in EGM-2 was added to each well, and an EC monolayer was formed by culturing the cells for 2 days. The EGM-2 was changed to the same medium (NG condition) or EGM-2 with 30 mM D-glu (HG condition), with or without AMA supplementation, at 5 h before ATP measurement. For exposure to hypoxia, a hypoxic culture kit (BIONIX, SUGIYAMA-GEN, Japan) was used to generate a hypoxic environment around the cells^42^. The amount of ATP extracted from the cells was measured using a firefly luciferase luminescence method with an intracellular ATP measurement kit, ver. 2 (IC2-100, TOYO B-Net, Japan). Luminescence was quantified using a luminometer (Lumat LB9510, Berthold Japan K. K., Japan), and the intracellular ATP concentration was calculated in reference to a calibration curve. The ATP amount, *P*, was divided by the amount of protein in the cell lysate as measured using the bicinchoninic acid method. The ratio *P*/*P*_NG_ over the amount of ATP, *P*_NG_, under the NG condition without AMA in the same plate was used to evaluate ATP production. The values *P* were measured in two to five wells, and those of *P*_NG_ were measured in three wells in the same plate. The final sample numbers were five and nine for *P* and *P*_NG_, respectively.

### Supplementary Information

Supplementary Information.

## Data Availability

The data presented in this study are available from the corresponding author upon reasonable request.
