# Supplementary material for: Hypoxia suppresses glucose-induced increases in collective cell migration in vascular endothelial cell monolayers
Source: Sci Rep. 2024 Mar 2;14:5164. doi: 10.1038/s41598-024-55706-1 (PMC10908842; doi:10.1038/s41598-024-55706-1)
Supplement: Supplementary file 1 — Supplementary Information. [file 41598_2024_55706_MOESM1_ESM.pdf]

SUPPLEMENTARY MATERIAL

"Hypoxia suppresses glucose-induced increases in collective cell migration in vascular endothelial cell monolayers" by Sone et al.

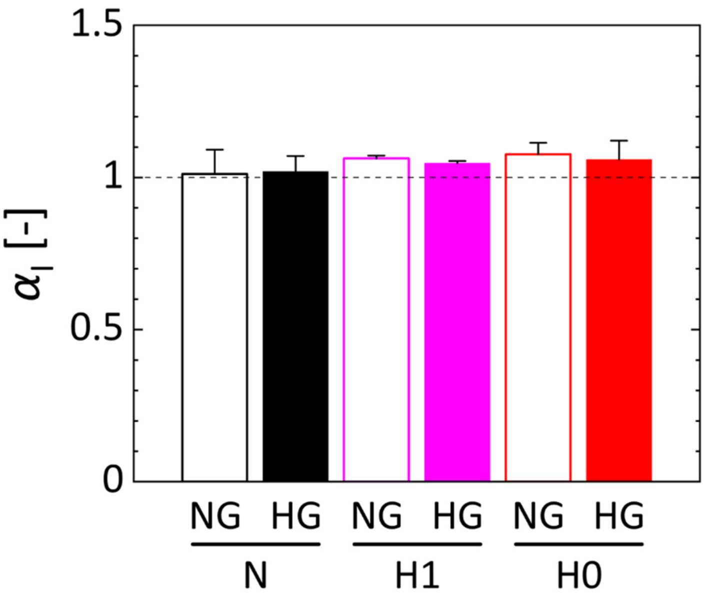

Fig. S1 Increase rate in cell numbers of HUVECs, α_I, during 5-h experiments under normal or high D-glucose conditions at 5.5 mM (NG) or 30 mM (HG) and three oxygen conditions with supply of gas mixture at 21%, 1% or 0% (N, H1 or H0, respectively). Error bars show the standard deviation. No significant difference was observed in migration speed by two-way ANOVA followed by Tukey's post-hoc test for multiple comparisons.

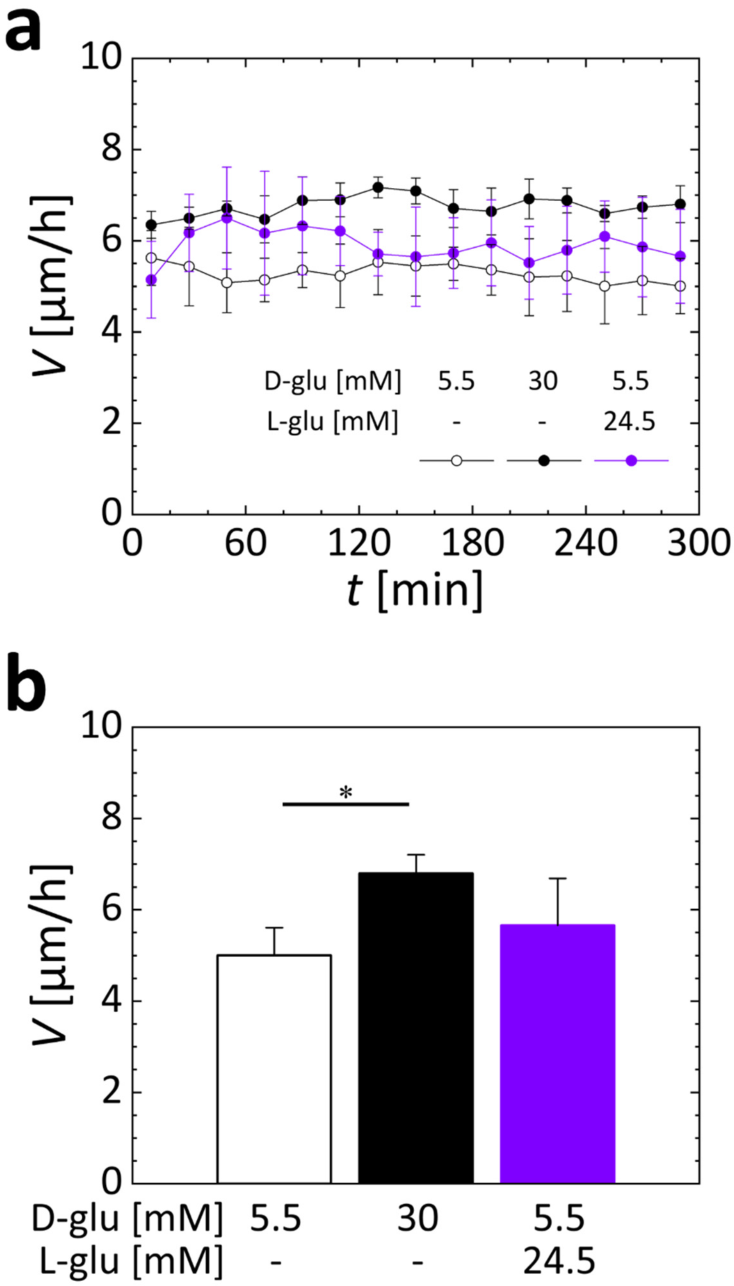

Fig. S2 Effect of osmotic pressure on collective cell migration of HUVECs. **(a)** Average migration speed, *V*, over time for 5 h, and **(b)** average migration speed at 5 h. Error bars show the standard deviation. Significant differences in migration speed between the different osmotic conditions were assessed by one-way ANOVA followed by Tukey's post-hoc test for multiple comparisons. $^{*}P < 0.05$.

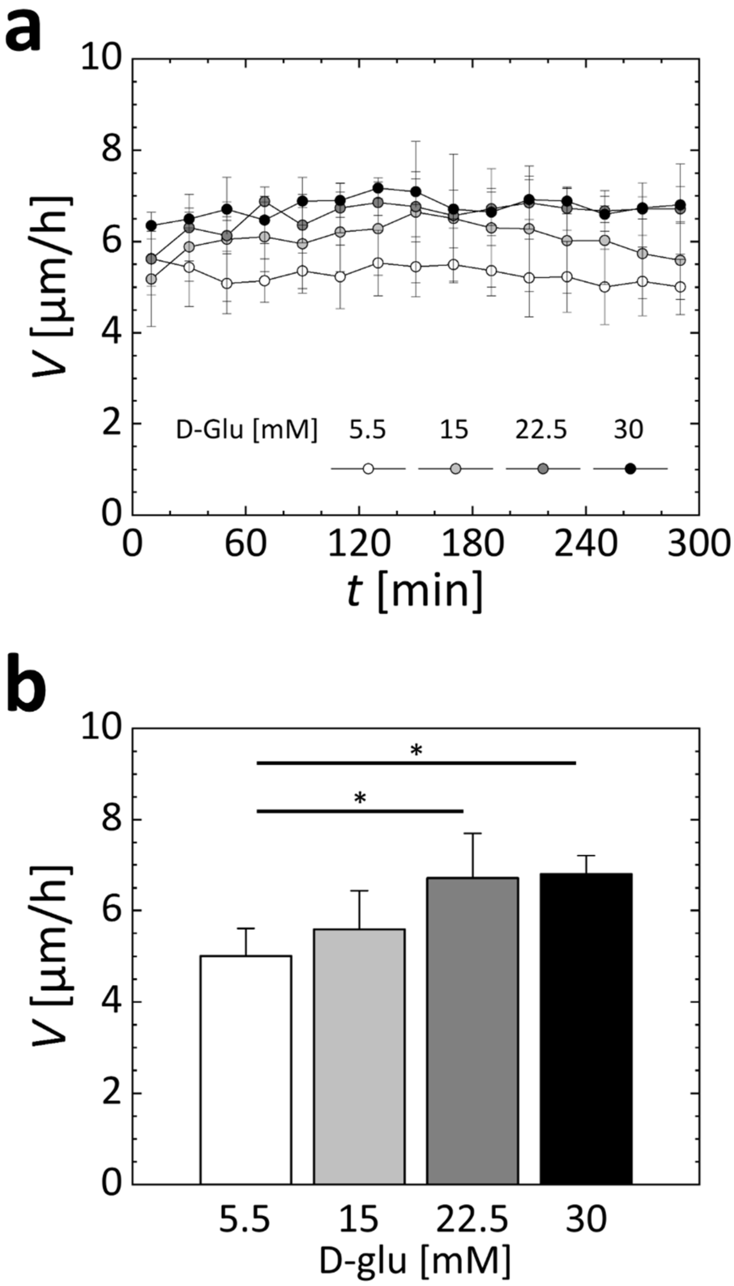

Fig. S3 Effect of D-glucose concentration on collective migration of HUVECs. **(a)** Average migration speed, V, over time for 5 h, and **(b)** average migration speed at 5 h. Error bars show the standard deviation. Significant differences in migration speed under four different glucose conditions (5.5, 15, 22.5, or 30 mM) were assessed by one-way ANOVA followed by Tukey's post-hoc test for multiple comparisons. $^{*}P < 0.05$.

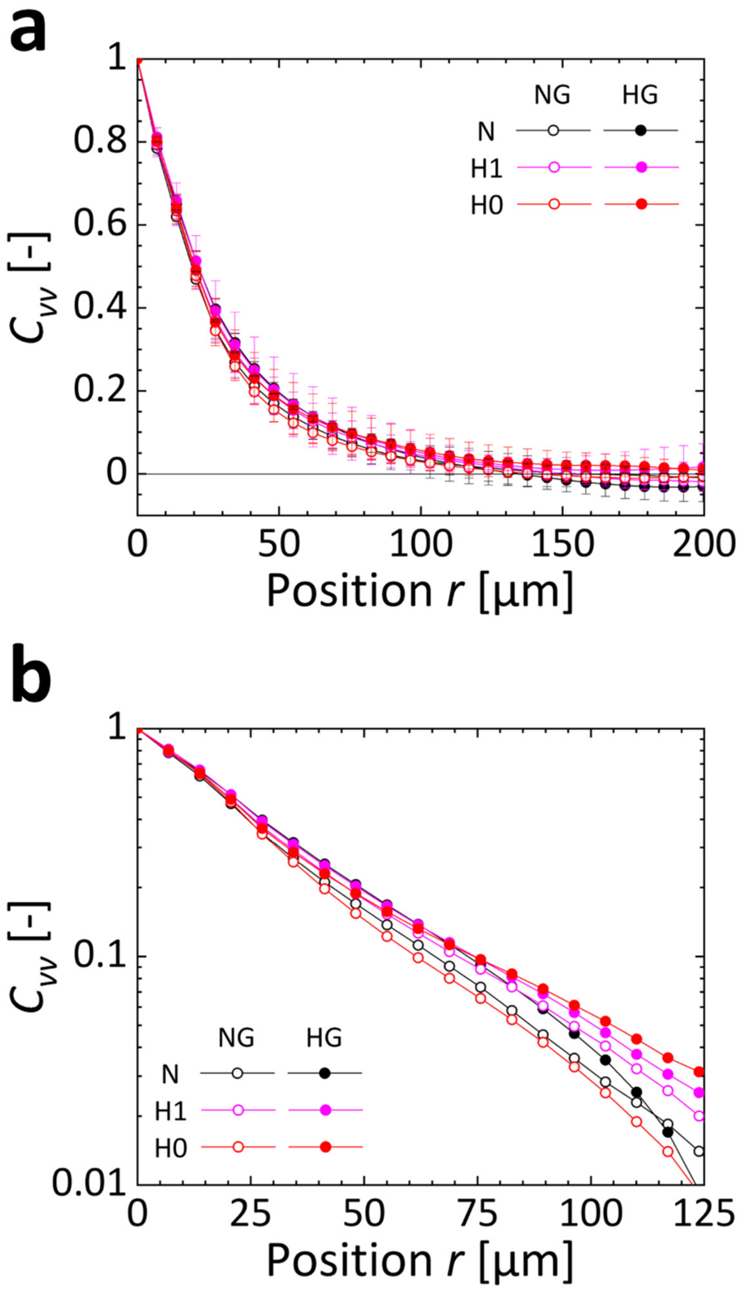

Fig. S4 Autocorrelation function of velocity fluctuation vectors C_{vv} of HUVECs under normal or high D-glucose conditions at 5.5 mM (NG) or 30 mM (HG) and three oxygen conditions with supply of gas mixtures at 21%, 1%, or 0% O_2 (N, H1, or H0, respectively): **(a)** the profile on a linear scale, and **(b)** the decay on a logarithmic scale. Error bars show the standard deviation.

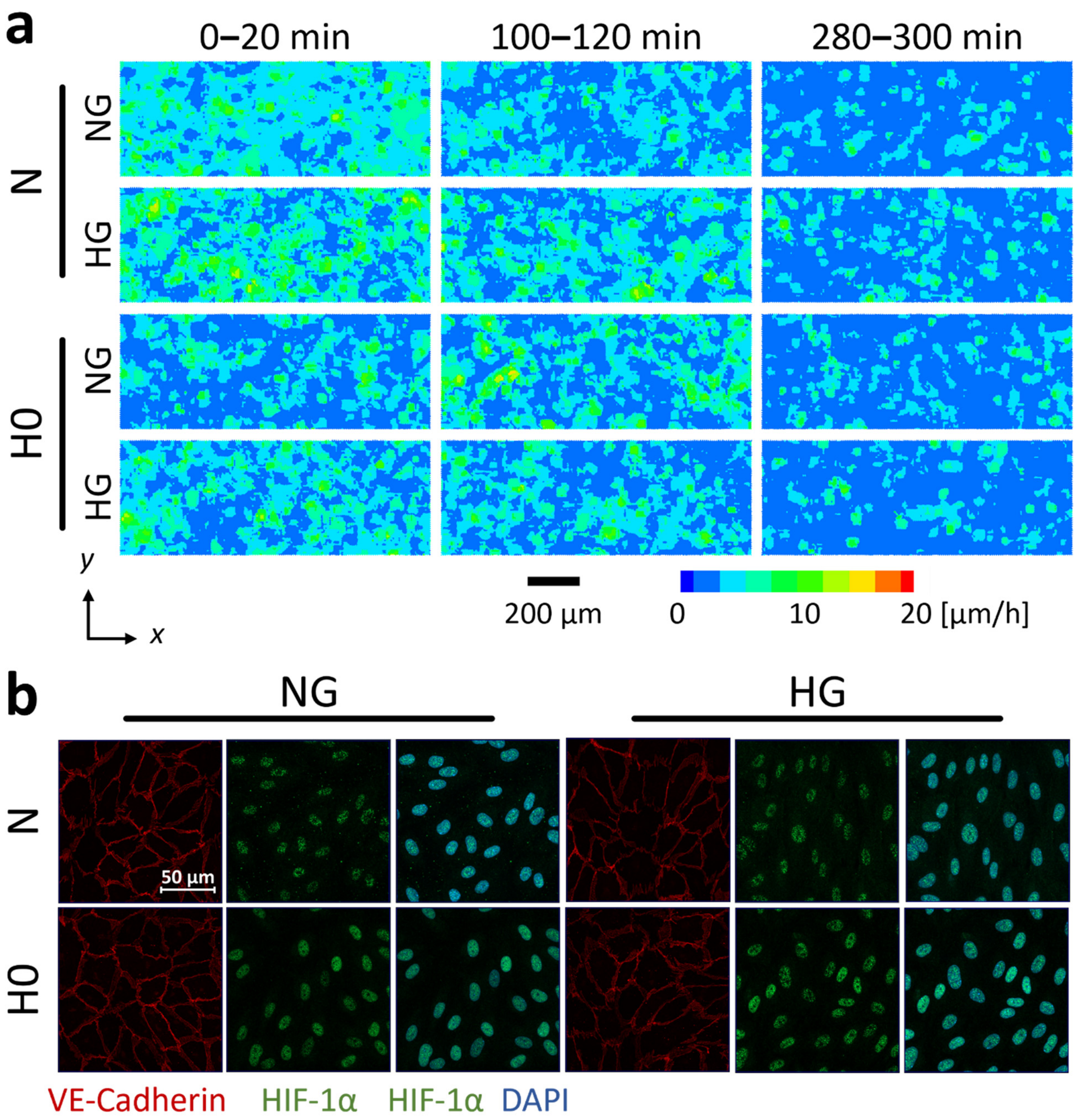

Fig. S5 Collective migration of HUVECs and intracellular protein expression and localization under normal or high D-glucose conditions of 5.5 mM (NG) or 30 mM (HG) and normoxic or hypoxic conditions (N or H0) with inhibition of mitochondrial electron transport by AMA. **(a)** Contour map of migration speed obtained by PIV analysis using phase-contrast microscopy images at 20-min intervals. **(b)** Representative images of maximum-intensity projections of confocal microscopy images of VE-cadherin (red), HIF-1α (green), and nuclei (DAPI, blue) in HUVECs relative to the *xy*-plane.

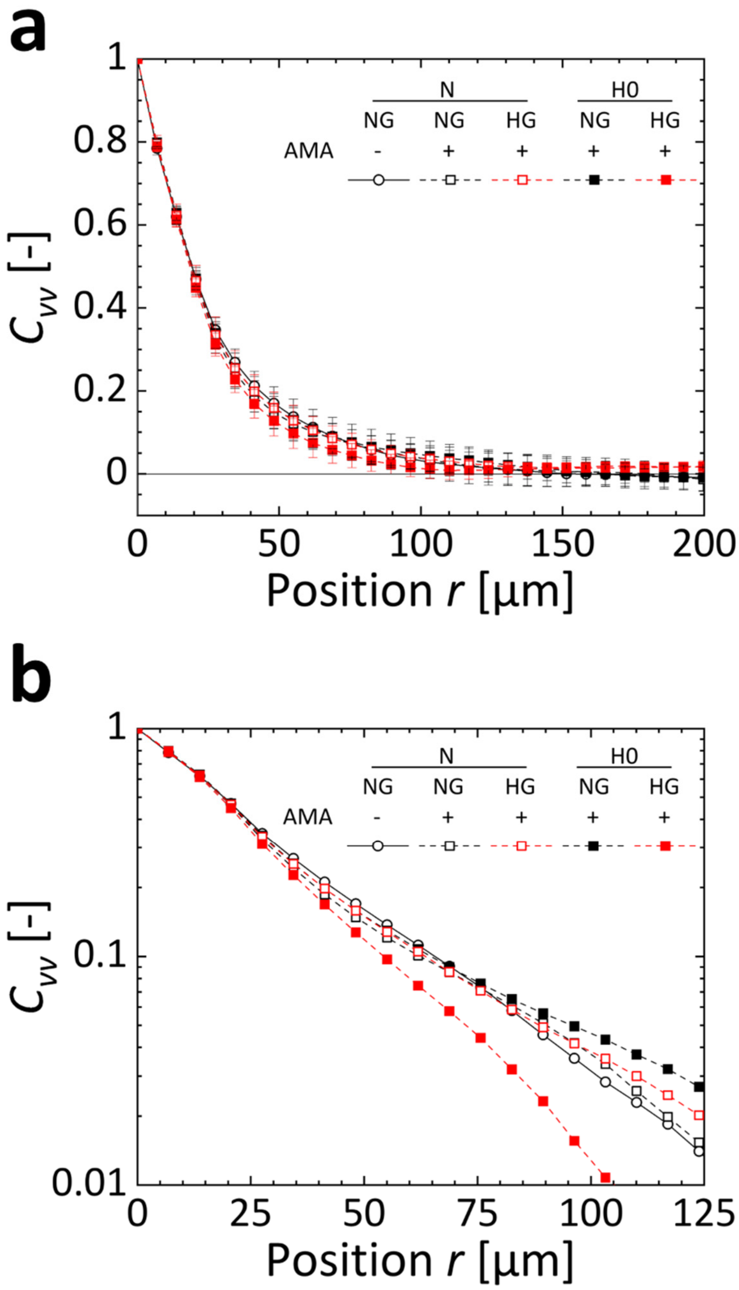

Fig. S6 Autocorrelation function of velocity fluctuation vectors C_{vv} of HUVECs under normal or high D-glucose conditions of 5.5 mM (NG) or 30 mM (HG) and normoxic or hypoxic conditions (N or H0) with inhibition of mitochondrial electron transport by AMA: **(a)** the profile on a linear scale, and **(b)** the decay on a logarithmic scale. Error bars show the standard deviation.

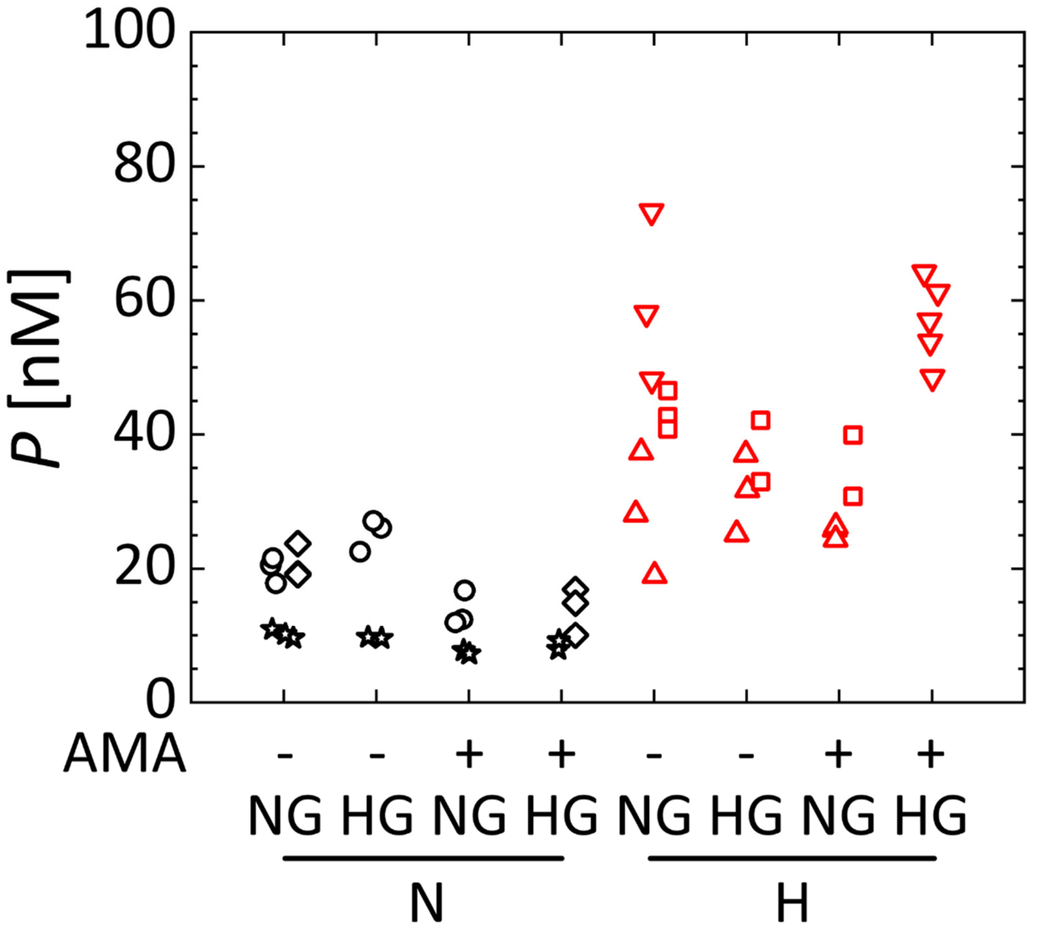

Fig. S7 Raw data of intracellular ATP in HUVECs under normal or high D-glucose conditions of 5.5 mM (NG) or 30 mM (HG) and normoxic (21% O_2, N) or hypoxic (0.1% O_2, H) conditions with inhibition of mitochondrial electron transport by AMA. The sample numbers for NG condition without AMA were nine, and those for the other conditions were five. The same symbol shape indicates the data obtained with the same well plate.
